# The Royal Naval Medical Service

**Published:** 1911-10-21

**Authors:** 


### THE ROYAL NAVAL MEDICAL SERVICE.

With commendable promptitude, the finding of the Durnford Committee has been crystallised into clear and concise regulations by Sir James Porter. It is probable that the new conditions will induce more men to present themselves, though the life of a surgeon in the Royal Navy does not compare all too favourably with the career of a surgeon under the War Office, the Colonial Office, or other Government Department.

Without preamble, the Regulations proceed to define the qualifications required. The limit of age is fixed at twenty-eight. Every candidate must be interviewed by the Director-General, who will decide whether he may be allowed to enter for the examination. About thirty commissions will be competed for annually, but the Admiralty reserves power to admit each year six Colonial graduates who may be specially recommended by Colonial universities. Candidates who have served in the Officers' Training Corps are credited at the examination with additional marks. A successful candidate is appointed an acting-surgeon and is required to pass through a course of six months' study; he will then be again examined and if he passes will be given a commission as surgeon. His position in the examination determines his seniority and considerable encouragement is given to candidates to hold ordinary House appointments in Civil Hospitals, for any successful candidate holding or about to hold the office of R.M.O. is allowed to retain or complete his period of office and the time so occupied counts for his general service in the Navy, though he may not be in receipt of Navy pay.

The entrance examination is a very ordinary one and is not likely to tax the capabilities of the average student, but the course of study and the examination prescribed during the probationary period cannot be taken lightly. In two months acting-surgeons are required to cover the following ground: Tropical medicine, bacteriology and clinical pathology, hygiene and skiagraphy. To the average man this will come as a shock. To get a fair knowledge of the first subject alone takes most men three or four months. It may be intended merely to gain a superficial survey of these subjects, but if so it is a course hardly to be commended. The above mentioned work will be done in the new school at Greenwich and the remainder of the probationary period as acting-surgeon will be spent at Haslar in the study of routine peculiar to the Naval surgeon. If the examination after this course is duly passed, a final one will follow towards the end of the first eight years of service. Then each officer will be required to attend a six months' course of post-graduate study and to pass an examination which will qualify for the rank of staff-surgeon. Having served eight years and passed the latter examination a surgeon is promoted to the rank of staff-surgeon. After eight years' service as staff-surgeon, he will be eligible for promotion to Fleet-surgeon. The pay of substantive rank has not been altered, but increments of pay will be given every two years during the time that the officer is on the active list. It is obvious that the increase of pay will not attract men. Still there is nothing to complain of in £1 a day after eight years' service, while staff-surgeons go up to £426 and Fleet-surgeons to £638, deputy-surgeons-general to £821 and surgeons-general to £1,300 a year. Though the pay does not bulk very large, the extras and allowances are considerable. It must be noted, too, that the feature which is most likely to attract from the purely material point of view is the generous scale of retired pay and allowances. After so short a period of service as four years a surgeon may retire with a gratuity of £500 with an additional £500 for each four years of service up to £2,000, while the retired pay of men who have served from twenty to thirty years varies from £1 to £2 per diem.

No doubt the younger members of the profession will agree that the new Naval Medical Service is worthy the attention of graduates seeking a career. Application should be made to the Admiralty for a full copy of the regulations. These set forth in detail the various provisions adopted under the new scheme, and exhibit an earnest endeavour to render the service worthy of men of the highest attainments.

